# Novel foods, food enzymes, and food additives derived from food by-products of plant or animal origin: principles and overview of the EFSA safety assessment

**DOI:** 10.3389/fnut.2024.1390734

**Published:** 2024-05-03

**Authors:** Gabriela Precup, Eleonora Marini, Panagiota Zakidou, Elisa Beneventi, Civitella Consuelo, Cristina Fernández-Fraguas, Esther Garcia Ruiz, Marcello Laganaro, Maura Magani, Agnieszka Mech, Estefania Noriega Fernandez, Irene Nuin Garciarena, Pablo Rodriguez Fernandez, Ruth Roldan Torres, Annamaria Rossi, Laura Ruggeri, Francesco Suriano, Ermolaos Ververis, Yi Liu, Camilla Smeraldi, Andrea Germini

**Affiliations:** ^1^European Food Safety Authority, Nutrition and Food Innovation Unit, Novel Foods Team, Parma, Italy; ^2^European Food Safety Authority, Food Ingredients and Packaging Unit, Food Enzymes Team, Parma, Italy; ^3^European Food Safety Authority, Food Ingredients and Packaging Unit, Food Additives and Flavourings Team, Parma, Italy; ^4^Department of Processing Technology, Nofima, Stavanger, Norway; ^5^Faculty of Veterinary, Department of Animal Production and Food Science, Food Technology Group, University of Zaragoza, Zaragoza, Spain; ^6^Department of Medical Biochemistry and Cell Biology, Institute of Biomedicine, University of Gothenburg, Gothenburg, Sweden; ^7^Department of Hygiene, Epidemiology and Medical Statistics, School of Medicine, National and Kapodistrian University of Athens, Athens, Greece

**Keywords:** agro-food by-products, safety assessment, novel foods, food enzymes, food additives

## Abstract

The European Union (EU) is committed to transitioning toward a circular economy model, with food waste being one of the areas to be targeted. To close the loop of food waste generated during food processing and discarded at the retail or consumption phases, research and innovation parties proposed to valorize agro-food by-products to produce novel foods and food improvement agents (food additives, food enzymes, and food flavorings). In the EU, the authorization of such novel foods and food improvement agents is governed by different regulatory frameworks. A centralized safety assessment by the European Food Safety Authority (EFSA) is the prerequisite for their authorization through the so-called Union Lists. Up to December 2023, EFSA published 45 scientific opinions on the safety of novel foods, food enzymes, and food additives derived from by-products of plant and animal origin. The current study illustrates examples of these by-products for the production of novel foods or food improvement agents and the data requirements behind their respective safety assessments conducted by EFSA. In this review, applications on novel foods, food enzymes, and food additives received by EFSA were screened and analyzed to find the common scientific requirements and differences in terms of the safety evaluation of such products. Various by-products (i.e., corncobs, coffee husks, spent grains of barley and rice, grape pomace, pumpkin peels, bovine whey, eggshells, shrimp heads, and animal organs or tissues) were described in the applications as being processed (extraction, physical treatments, and chemical and enzymatic reactions) to obtain novel foods and food improvement agents. The heterogeneity and complexity of these products emphasize the challenge of their safety assessment, depending on the characteristics of each product. However, as this study shows, the scientific requirements underpinning their safety do not differ substantially in the different regulated product areas considered, with similar information needed to assess their safety in terms of identity, production process, compositional characterization, proposed/intended uses and exposure assessment, toxicological information, and allergenicity data. Additional nutritional information and data on the history of use are required in the case of novel foods.

## Graphical abstract

Schematic representation of the principal stages of the regulatory framework underlying the authorization of novel foods, food enzymes, and food additives derived from agro-food by-products.

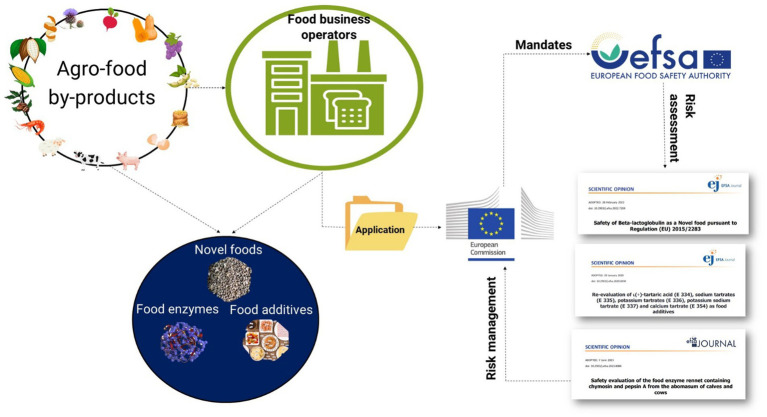

## Introduction

The European Commission (EC) aims to position Europe as the world’s first climate-neutral continent by 2050, alongside transitioning to a circular economy model. This plan requires well-defined strategies to address current challenges, including demographic changes, depletion of natural resources, climate change impact, malnutrition, and the increasing prevalence of non-communicable diseases (1). The European Green Deal is the overarching framework outlining transformational changes required to achieve these objectives, notably through its Farm to Fork strategy designed to cultivate a fair, healthy, and environmentally sustainable food system (2).

A major focus of the European Union (EU) is to reduce food waste, which is in line with the Sustainable Development Goal Target 12.3, aiming to halve *per capita* food waste in the EU by 2030. The EC’s revised EU waste legislation emphasizes initiatives such as implementing a common EU methodology for measuring food waste consistently, establishing the EU Platform on food losses and food waste, clarifying legislation related to waste, food, and feed, facilitating food donation, and improving understanding of date marking (3).

Globally, according to FAO, approximately one-third of all food produced for human consumption is lost or wasted from the post-harvest stage up to, but excluding, the retail stage, which accounts for 14% of all food produced (4). In the EU, over 58 million tons of food waste are generated annually, accounting for approximately 10% of the total food made available to EU consumers (at retail, food services, and households) (5).

Agro-food by-products of both plant (e.g., pulp, peel, seeds, pomace, husks, pods, stems, roots, tuber and oil crops residues, cereal bran, hulls, and spent grain) and animal origins (e.g., blood, bones, fatty tissues, internal organs, meat trimmings, and skin) are mainly generated during primary production or processing stages. These by-products could serve as sources of dietary fibers, lipids, proteins, and other nutrients or could be utilized as raw materials (6–12). One of the solutions proposed in the field of research and innovation is the valorization of food by-products and side streams for the manufacturing of food and feed. This approach could offer various environmental and economic advantages (6–12).

The scientific literature highlights the potential of various food by-products in yielding specific components (such as carotenoids, oligosaccharides, organic acids, polyols or polyphenols, enzymes, and peptides) and contributing to the production of various food products (for instance, fermented beverages, dairy and meat products, and bakery formulations) (7, 8, 13–18). At the same time, several challenges have also emerged from the valorization of food by-products. These challenges mainly revolve around the stability of by-products, low energy efficiency, and high costs associated with the extraction and downstream processes. Moreover, concerns arise regarding using extraction solvents not permitted for food processing, the presence of potential pathogens, and other physical, microbiological, and/or chemical contaminants in the food by-products, hazards that may pose a risk to human health (8, 19). It is worth noting that chemical and microbiological agents and practices on the hygiene of foodstuffs are governed by specific EU regulations.

Therefore, the integration of food by-products into the food chain requires a thorough evaluation of their safety and of the most suitable upcycling methods for the manufacturing process before being placed on the market. In the EU, the use of food by-products falls under distinct regulatory frameworks, depending on their intended use. For instance, if a food or food ingredient has not been used for human consumption to a significant degree within the European Union before 15 May 1997, it can be considered a novel food (NF), falling under Regulation (EU) 2015/2283^1^ on NF. In the case of food by-products used in the production of food additives (FA), Regulation (EC) No 1333/2008^2^ lays down rules on FA used in food products. The FA are defined as any substance not normally consumed as a food in itself and not normally used as a characteristic ingredient of food, whether or not it has nutritive value, the intentional addition of which to food for a technological purpose in the manufacture, processing, preparation, treatment, packaging, transport or storage of such food results, or may be reasonably expected to result, in it or its by-products becoming directly or indirectly a component of such foods. Permitted FA in the EU are identified by an E number, are subject to evaluation, and, to be lawfully marketed, must comply with certain standards (e.g., of purity, including microbiological criteria) laid down in Regulation (EU) No 231/2012. Food by-products can also serve as a source of food enzymes (FE), which fall under Regulation (EC) No 1332/2008.^3^ This regulation concerns FE and FE preparations used as processing aids, and FE performing a technological function in food. FE are biological catalysts derived from animals, plants, microorganisms, or products thereof, added to food for a technological purpose, whereas FE preparations are formulations of FE and substances incorporated to ameliorate the food enzyme composition (excipients) (i.e., for dilution or storage). Irrespective of the substance or food products and improvement agents to be assessed, the principles of the safety assessment are identical among NF, FE, and FA; however, the data requirements stemming from the respective regulations and at the basis of the RA may be different. This review aims to provide an overview of the scientific requirements for the safety assessment of NF, FE, and FA derived from food by-products, drawing upon practical examples from published scientific outputs. The commonalities and differences in terms of safety assessment of the respective food products and food improvement agents are also outlined.

## Overview of the applicable regulatory frameworks for novel foods, food enzymes, and food additives derived from by-products

An authorization procedure is required before the EU market authorization of an NF and an FA to maintain an FE on the EU market or in case of changes to the conditions of use or changes in the manufacturing process of such products. This includes a risk analysis that encompasses three main areas: risk assessment, risk management, and risk communication. Food business operators (FBOs) who intend to place such products on the EU market must submit an application to the EC. EFSA may be tasked by the EC to carry out the risk assessment (RA) of the technical dossiers submitted in the context of the application and provide scientific advice to support risk managers (EC, European Parliament, Member States). The RA is an independent scientific process consisting of four pillars: (i) hazard identification, (ii) hazard characterization, (iii) exposure assessment, and (iv) risk characterization. Whereas hazard identification intends to identify and quantify the hazard, hazard characterization evaluates the adverse health effects of the identified hazards and, when possible, translates them to safe levels. The exposure assessment aims to qualitatively and/or quantitatively evaluate the likely intake of biological, chemical, and physical agents via food, as well as exposures from other sources, if relevant. Ultimately, the outcomes of the hazard identification, characterization, and exposure assessment are integrated into the risk characterization, estimating the final risk in a given population. Although the four guiding steps of the RA are common among NF, FE, and FA, the data requirements necessary to conduct the RA can vary due to the different regulations and the nature of the product under assessment.

The RA of NF, FE, and FA is conducted by scientific groups of external experts, namely the EFSA Panel on Nutrition, Novel Foods and Food Allergens (NDA), the Panel on Food Additives and Flavourings (FAF), and the Panel on Food Contact Materials, Enzymes, and Processing Aids (CEP), respectively. These panels are supported by specific Working Groups and EFSA scientific officers. The outcome of the RA of the technical dossiers is captured in a scientific output, which is to be adopted by the relevant Panel within 9 months of receiving a valid application. The adopted scientific output is published in the EFSA Journal. The European Commission and the EU Member States consider the EFSA’s safety assessment and draft an implementing act, deciding whether to authorize or not the NF, FE, or FA.

### Novel foods regulatory framework

In 2018, the implementation of Regulation (EU) 2015/2283, repealing and replacing Regulation (EC) No 258/97,^4^ designated EFSA as the centralized EU entity responsible for carrying out the RA of NF. Under this regulation, FBOs are responsible for verifying with their national competent authorities whether the product they intend to market falls within the NF categories defined by the regulation and, therefore, would require an application for marketing authorization. Member States may be consulted to support this decision, following the procedure laid down in Commission Implementing Regulation (EU) 2018/456.^5^ Upon confirming the validity of the application with respect to the requirements laid down in Regulation (EU) 2015/2283 on NF, the EC makes the technical dossier available to the Member States and may mandate EFSA to carry out the RA in accordance with Article 10 of the respective regulation.

Applicants are recommended to comply with the main scientific requirements outlined in EFSA’s Guidance on the preparation and presentation of an application for authorization of an NF in the context of Regulation (EU) 2015/228 (20) for the preparation of their technical dossier. EFSA’s mandate in the RA of NF refers to the safety assessment of the product for the general EU population or for specific population groups, including an evaluation of whether the product could be nutritionally disadvantageous for the consumer. The EC adopts the authorizing implementing act only after the Standing Committee on Plants, Animals, Food and Feed (PAFF Committee) has granted a positive output. Afterward, the implementing act is published in the Official Journal, and the NF is included in the Union List of Authorized Novel Foods,^6^ together with its specifications, conditions of use, specific labeling requirements, and/or post-market monitor requirements (if necessary), in line with Regulation (EU) 2017/2470.

### Food improvement agents (food additives, food enzymes, and food flavorings) regulatory frameworks

In the case of food improvement agents FA, FE, and food flavorings (FF), a Common Authorization Procedure is described in Regulation (EC) No 1331/2008^7^ and implemented in Regulation (EU) 234/2011.^8^ These regulations introduced a harmonized, effective, and transparent authorization procedure within the EU to facilitate the free movement of food while ensuring consumer health.

The EC, an EU country, or an interested party can initiate the procedure through an application for updating the EU list of authorized FA, as per Commission Regulation (EU) No 1130/2011,^9^ while Commission Regulation (EU) No 231/2012^10^ lays down specifications for the FA.

Before EFSA, other bodies, such as the Scientific Committee on Food (SCF) and the Joint FAO/WHO Expert Committee on Food Additives (JECFA), were involved in the RA of FA. EFSA has three main tasks in relation to FA: evaluating the safety of new FA or proposed new uses of existing FA before they can be authorized for use in the EU, re-evaluating all FA permitted for use in the EU before 20 January 2009, and responding to *ad-hoc* requests from the EC to review certain FA in the light of new scientific information and/or changing conditions of use.^11^ The data requirements for applications supporting the authorization of a new FA or modifications to an already authorized FA are highlighted in the EFSA’s Guidance for submission for FA evaluations (21). In the case of the re-evaluations of the FA, EFSA publishes relevant calls for data to address data gaps and receives new information from the interested business operators (IBO), in line with Regulation (EU) No 257/2010.^12^

FE are still considered and evaluated as a type of food additive by JECFA today. In the EU, before January 2009, acronym FE other than those used as food additives were not regulated or were regulated as processing aids under the regulatory frameworks of the Member States. Until 2008, only two Member States (France and Denmark) in the EU performed safety assessments on enzymes for food uses. On 20 January 2009, Regulation (EC) No 1332/20081 on food enzymes entered into force. This regulation applies to enzymes that are added to food to perform a technological function in the manufacture, processing, preparation, treatment, packaging, transport, or storage of such food, including enzymes used as processing aids.

All FE currently on the EU market and new FE must undergo a centralized safety evaluation by EFSA and approval by EC. Authorization to the use of an FE is granted upon three conditions: the proposed use does not represent a risk for consumers; the relevance of the technological use is verified; and consumers are not misled by the FE use. The scientific requirements for their assessment, in accordance with Regulation (EC) No 1331/2008, are outlined in EFSA’s Scientific Guidance for the submission of dossiers on FE (22). Implementing Regulation 562/2012,^13^ together with the Scientific Guidance, provides the requirements for derogating toxicological studies for the safety assessment of an FE derived from edible sources and their by-products (see section 3.2.5). An EU community list of FE, including all the authorized FE, is currently under preparation. Thus, for the time being, the decision to place FE for food processing on the market is subject to the ruling of national legislation.

## Methodology

Published scientific outputs (e.g., scientific opinions, technical reports, and re-evaluations) until December 2023 on the RA of NF, FE, and FA derived from by-products were retrieved from the EFSA’s Journal.^14^ The corresponding applications were also considered.

The search keywords included “Novel Foods,” “Traditional Food,” “Food Additives,” and “Food Enzymes.” The resulting outputs were further screened to identify NF, FE, or FA derived from animal and plant by-products. By-products derived NF, FA, and FE from microbial fermentations were not considered for the purpose of the present article since such information might not be provided by the FBOs in line with the requirements of the specific regulatory frameworks. Where applicable, data on the identity of the source, manufacturing process, compositional data, proposed uses, dietary exposure, toxicological information, and allergenicity were collected and collated in a tabulated form.

## Results

### Applications of novel foods, food enzymes, and food additives derived from by-products

A total of 45 published scientific outputs on the safety of NF, FE, and FA derived from by-products of plant and animal origin were retrieved from the EFSA’s Journal. Various by-products were used in their production process (Table 1).

**Table 1:** Agro-food by-products used for the production of novel foods, food enzymes, and food additives.

Origin	Novel Foods	By-Products	Food Additives	By-Products	Food Enzymes	By-Products
Plant Origin	Cacao fruit pulp (*Theobroma cacao* L), including the juice made from the cacao fruit pulp	Cocoa pulp	L(+)-Tartaric acid	Tartar, lees of wine, and/or grape pomace	Peroxidase	Soybean (*Glycine max*) hulls
	Xylo-oligosaccharides	Corn cobs	Vegetable carbon	Vegetable materials such as wood, cellulose residues, peat, coconut, and other shells	Phytepsin (x4)	Cardoon flowers of *Cynara cardunculus*
	Cherry pulp (or dried cherry pulp) from *Coffea arabica* L. and *Coffea canephora* Pierre ex A. Froehner (x2)	Coffee cherry pulp	Soybean hemicellulose	Okara – soybean fiber	L-Ascorbate oxidase	Fruit peel of *Cucurbita pepo* and *Cucurbita moschata* (squash, pumpkins)
	Coffee husk (Cascara)	Coffee husk	Neohesperidine dihydrochalcone	Peels of bitter orange and grapefruit		
	Partially hydrolyzed proteins from spent barley and rice	Barley and rice from the mash step of beer production	Inline figure 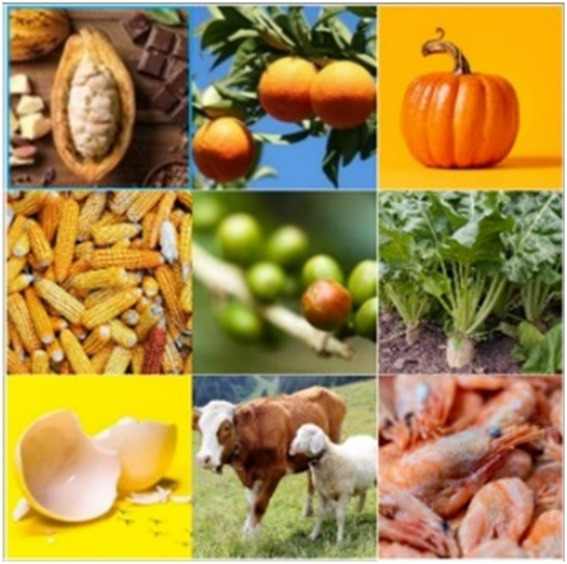		Protease complex containing Trypsin, (x6)	Porcine pancreas
	Rapeseed powder from *Brassica rapa L.* and *Brassica napus L.*	Seeds of non-genetically modified (non-GM) cultivars of *Brassica rapa L*. and *Brassica napus L.*			Phospholipase A2	Porcine pancreas
Animal origin	Shrimp peptide concentrate	Shrimp shells and heads			Animal Rennet (x9)	Abomasum of cows, goats, and sheep (adults and suckling)
	Egg membrane hydrolysate	Egg membrane			Triacylglycerol lipase	Pregastric tissues (gullet) of cattle, goats, and sheep
	Bovine milk basic whey protein isolate; Beta-lactoglobulin (BLG)	Whey			Thrombin	Cattle or pigs blood
	Galacto-oligosaccharides (x3)	Milk lactose or sweet whey			Catalase (x2)	Porcine liver

NF derived from by-products accounted for 15 outputs pursuant to Regulation (EU) 2015/2283. Eight of these NF were derived from by-products of plant origin: xylo-oligosaccharides (corncobs), cherry pulp from *Coffea arabica* L. and *Coffea canephora* Pierre ex A. Froehner, cacao fruit pulp (*Theobroma cacao* L), coffee husk, spent grains from barley (*Hordeum vulgare*) and rice (*Oryza sativa*), betaine (sugar beets molasses, vinasses, or betaine glycerol), and rapeseed powder (*Brassica rapa* L. and *Brassica napus*). Seven NF were instead derived from by-products of animal origin: shrimp peptide concentrate, egg membrane hydrolysate, beta-lactoglobulin, bovine milk basic whey protein isolate, and galacto-oligosaccharides (23–37).

Regarding FA, outputs of four different FA derived from by-products of plant origin re-evaluated by EFSA were retrieved, referring to tartaric acid (E 334) (tartar, lees, and grape marc of wine and/or grape pomace), vegetable carbon (E 153) (peat, wood, cellulose residues, coconut shells, or other shells), soybean hemicellulose (E 426) (okara - soybean fiber), and neohesperidine dihydrochalcone (E 959) (peels of bitter orange grapefruit) (38–41).

In the case of FE, 26 EFSA outputs were retrieved referring to 6 FE derived from by-products of plant origin: an L-Ascorbate oxidase (extracted from the fruit peel of squash and pumpkins *Cucurbita pepo* and *Cucurbita moschata*); a peroxidase (from soybean *Glycine max*, hulls); and 4 phytepsins (from the Cardoon flowers of *Cynara cardunculus*). Twenty outputs referred to FE obtained from animal by-products: 9 animal rennet (from the abomasum of cows, goats, and sheep); 2 catalases (from the porcine liver); 6 protease complexes containing trypsin, a phospholipase A2 (from the porcine pancreas); a thrombin (from cattle’s or pigs’ blood), and a triacylglycerol lipase (from the pregastric tissue of cattle, goat, and sheep) (42–67).

## Overview of the common key scientific requirements for the safety assessment of novel foods, food enzymes, and food additives derived from by-products

### Identity of the source and/or the product

**Figure 1:**
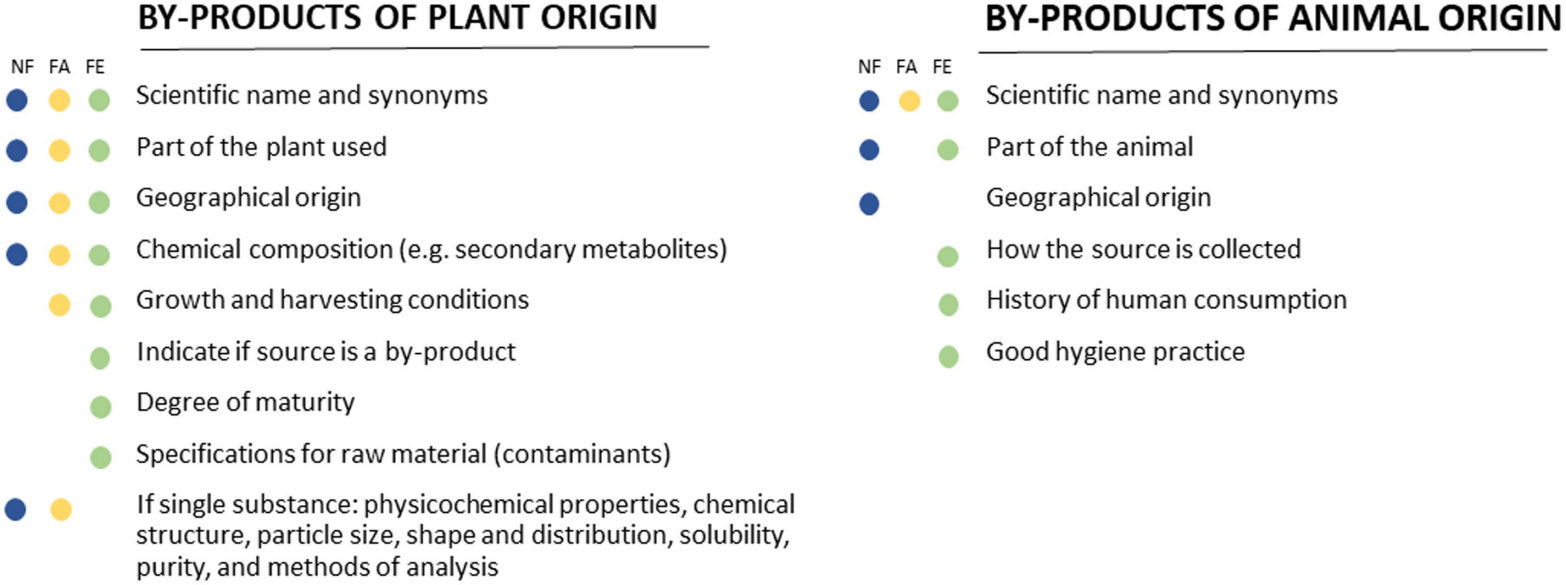
Schematic overview of the scientific requirements on the identity of novel foods, food enzymes, and food additives derived from by-products.

The requirements regarding the identity of the NF and FA, or the source of the FE of plant origin, include the scientific name according to the international codes of nomenclature; synonyms (botanical name), the specific parts of the plant used (e.g., root, leaf, and seed) and the geographical origin (continent, country, and region). For enzymes derived from plants, the applicant should also indicate if the plant source is a by-product of former processes and specify the stage of maturity at which the selection is carried out. Additionally, the growth and harvesting conditions (wild or cultivated, cultivation practices, and time of harvest concerning both season and stage of the plant growth) are requested in the case of FA and FE (Figure 1).

The quality of the raw material, information on the chemical composition of the plant-derived FA and NF, i.e., the concentration of characteristic constituents (e.g., flavonoids and terpenoids) or in the case of enzymes derived from non-edible plant fractions, the capacity of the plant source to produce secondary metabolites that could be harmful to humans, should be provided. For instance, some *Cucurbita* spp. can produce a group of cytotoxic steroids referred to as cucurbitacins. However, the *Cucurbita* spp. selected for human consumption do not produce these steroids or do so at very low levels as their strong bitter taste would make them inedible (66). Regarding the powder produced from the seeds of non-genetically modified (non-GM) cultivars of *Brassica rapa* L. and *Brassica napus* as an NF, the presence of plant secondary metabolites of concern (erucic acid and glucosinolates) was analyzed (33). Furthermore, the maximum levels for microorganisms and possible contaminants, including heavy metals, mycotoxins, pesticide residues, and polycyclic aromatic hydrocarbon (PAH) residues, should also be documented. Following Regulation (EC) No 396/2005^15^ residual pesticides in edible animal and plant products should be analytically quantified and considered acceptable only if within legally permitted levels. Soybean hulls used for the extraction of the FE peroxidase were regularly controlled for contaminants (i.e., heavy metals, pesticides, and mycotoxins), as shown in the certificate of analysis provided for a batch of soybean hulls. The source of the FE was, therefore, considered safe for consumption (62).

In the case of plant by-products used to extract single substances, the main elements to be considered to document the identity refer to the physicochemical properties (e.g., appearance, melting point, boiling point), chemical structure(s), solubility data in water and other common solvents, particle size, shape, and distribution and purity value. L(+)-tartaric acid as FA obtained from tartar, lees of wine, and/or grape pomace is a colorless or translucent crystalline solid or white crystalline powder, very soluble in water, with a purity ≥99.5% on the anhydrous basis. However, it can be contaminated by heavy metals such as lead and mercury, as well as by oxalates. Its derived products have a purity of more than 98% and may be contaminated by the same impurities, as well as arsenic (39).

For single substances such as FA, information on the proposed FA and its main components is given in terms of chemical structure(s) and physicochemical properties. The specifications of an additive define the requirements concerning the identity, the purity, and the limits of any impurity present in the additive, also indicating the appropriate methods of analysis.

For NF derived from by-products of animal origin, it is essential to provide taxonomic information on the biological source, including details on family, genus, species, subspecies, and variety, according to international nomenclature codes. Moreover, applicants should specify the organ, tissue, or part of the organism sourced. For example, shrimp peptide concentrates as an NF was obtained from the shells and heads of shrimps of the species *Pandalus borealis*, serving as a by-product of the production of cooked and peeled shrimps (34). For animal-derived FE, the genus and species of the FE source should be identified using the most recently accepted nomenclature. Applicants should describe how the animal product or tissue has been collected and, according to Regulation (EU) No 2015/1162,^16^ indicate whether the source is fit for human consumption. For instance, as specified by EC Regulation 1774/2002,^17^ the abomasum of calves and cows is an edible offal and considered suitable for consumption. The raw material (animal tissue) and the procedures of enzyme extraction should comply with the inspection requirements of official health authorities and follow the Food Hygiene Regulation (EC) No 852/2004^18^ and Regulation (EC) No 853/2004.^19^ Proof of the absence of any infectivity risk should be provided, along with the methods used. For example, the animal source of the protease FE complex containing trypsin, extracted from porcine pancreas, is collected following the EU regulations mentioned above and derived only from animals approved for human consumption (44). The veterinarians in charge of the slaughtering certified that the FE source is free from disease (such as African and classical swine fever, foot and mouth disease, and swine vesicular disease). Possible contaminating parasites (such as *Trichinella spiralis*, *Taenia solium*, and *Toxoplasma gondii*) and bacteria (such as *Yersinia enterocolitica*, *Listeria monocytogenes*, and *Salmonella* spp) is removed by freezing and filtering the meat by-product (44). In cases where equivalence to the EU hygiene standards is lacking, or no information on the veterinary inspection of carcasses is provided, the body of evidence fails to meet the EFSA requirements, and no conclusion on the safety of the FE can be drawn (46).

### Production process

The impact of the production process on the composition of NF, FA, and FE must be investigated, and a detailed description is required for the RA, including information on (i) raw materials, processing aids, food-contact materials and potential by-products, and impurities or process-related contaminants (e.g., solvents and reagents); (ii) unit operations and operational conditions for upstream and downstream processing, production flow charts, and packaging and storage practices; and (iii) measures implemented for production control and quality and safety assurance (Good Manufacturing Practice (GMP), Hazard Analysis Critical Control Points (HACCP), and International Organization for Standardization (ISO)). In the case of NF, the characterization of novel aspects of the production process (if not used for food production within the Union before 15 May 1997) must also be described.

Hazards may be introduced through the production process of the FE, as a number of processing aids could be used during the main extraction process of the enzyme and the subsequent downstream processes (e.g., removal of biomass and concentration of the enzyme liquor). Enzymatic pre-treatments could also be applied before the extraction of the enzyme from the plant or animal source. For the extraction of FE from animal- and plant-derived by-products, only permitted organic solvents can be used. As an example, for the safety assessment of catalases extracted from porcine liver, the CEP Panel concluded that the use of this enzyme may present a risk for consumers due to the use of a non-permitted solvent during the production of the FE (Directive 2009/32/EC)^20^ (60, 61). In the case of FE preparations, formulation ingredients are intentionally added to enable storage, to increase shelf-life, or as a means of standardization. In these situations, quantitative data on all added excipients is required. When the FE is immobilized or encapsulated, additional information on support/encapsulating materials, crosslinking agents, and/or other substances used in immobilization is requested, as they may be potentially hazardous.

In case of significant changes in the production process, for instance, when a new method is employed, different starting materials or formulations from conventional bulk to nanoscale dimensions are used, or the manufacturing process is taking place in several production sites, the differences should be reflected in the description of the manufacturing process. It is noteworthy that confidentiality in the detailed description of the production process is often requested by NF applicants following Article 23 of the Regulation (EU) 2015/2283 on NF.

NF are produced under quality and safety assurance systems, in line with GMP and HACCP principles, and typically in compliance with the Food Safety System Certification (FSSC) 22000. The production of FE should meet food safety management system principles (Commission Notice C/2016/4608)^21^ in agreement with the Food Hygiene Regulation (EC) No 852/2004.^22^ For FE that are subjected to national regulations and manufactured outside the EU, an equivalent certification should confirm the hygiene requirements. The NF derived from by-products can be broadly categorized as produced by (i) enzymatic reaction; (ii) chemical reaction or physical separation; and (iii) drying or freezing, while in the case of food improvement agents, the manufacturing process mostly encountered was chemical reaction and/or physical separation, or it was not disclosed by the applicant. In a few cases, food additives (e.g., soy lecithin in beta-lactoglobulin) or bulking agents (e.g., maltodextrin in xylo-oligosaccharides (XOS)) are added into the final formulation of the NF for technological purposes. The end-products are commercialized in powder form (i.e., Galactooligosaccharides (GOS), as (concentrated) syrups/liquids (cocoa pulp), crystals (anhydrous or monohydrate betaine), or dried whole products (coffee husk (cascara) and coffee cherry pulp)) (23–27, 29–32, 36).

When microorganisms are used in the production of the NF or FA (i.e., betaine as NF extracted from sugar beet vinasses^23^ obtained from fermentation of sugar beet molasses with *Saccharomyces cerevisiae*), the microbial production strains should be characterized according to the EFSA Guidance on the characterization of microorganisms used as feed additives or as production organisms (67) and EFSA (2021) statement (68). The safety of the FE used in the manufacture of the NF is subject to the provisions of Regulation No1332/2008 and, therefore, outside the scope of Regulation (EU) 2015/2283 on NF. However, if food enzymes used in the production of the NF or FA are not assessed by the EFSA CEP Panel at the moment of evaluating the NF or the RA is still in progress, additional data could be requested to establish the safety of the NF or FA in line with relevant EFSA guidance documents (20–22).

The principle behind the manufacturing processes employed for the production of these food products and improvement agents, including details on potential hazards and their assessment, are presented in Supplementary Table S1.

### Compositional characterization and specifications

The compositional characterization, in terms of chemical structure(s) and physico-chemical properties, is critical for the safety assessment of NF, FE, and FA derived from by-products, offering a comprehensive understanding of their attributes and potential hazards that might occur.

Due to the heterogenicity and complexity of NF, qualitative and quantitative data on physicochemical, biochemical, and microbiological characterization should be provided from experimental analysis and further substantiated by data from the scientific literature in a structured way by following the requirements from the EFSA Guidance document (20). The experimental data should be representative of the final product, and if there is variability among samples, a rationale should be provided. The certificates of analysis and information on the accreditation of laboratories are also required, including information on the analytical methods used for the analyses. Validated methods should be used, preferably nationally or internationally recognized, with suitable limits of detection and quantification. In-house methods are also accepted, but a full description of the methodology, along with its validation procedures, must be provided. In the case of FA, all methods of analysis employed should be presented and described in a separate section, following the requirements from the respective Guidance document (21).

From a compositional perspective, NF can be single substances, simple mixtures, complex mixtures, or whole foods, making their complete characterization challenging. When the NF is whole food, a proximate analysis should be conducted, along with the provision of the percentage of the potentially unidentified fraction (components that could not be characterized and/or quantified) should be provided. When the NF is characterized by a main group of components, its complete characterization is needed. For instance, for the safety assessment of the egg membrane hydrolysate, the main component was represented by proteins, and the total amino acid composition of the NF was requested by EFSA to be compared against the native egg membrane (35). Differences in the content of amino acids were observed, which might be owing to the hydrolyzation step that occurs during the manufacturing process of the NF. Microbiological characterization is also required for NF and FE. For the safety evaluation of the FE phospholipase A2 from porcine pancreas, EFSA could not conclude on its safety because not all the microbiological requirements for purity were met (no evidence of the absence of hepatitis E virus, *Salmonella* spp., *Campylobacter,* and *Escherichia coli* was provided), and the information on the FE chemical characterization and manufacturing process was incomplete (no equivalence to Food Hygiene Regulation (EC) No 853/2004 was submitted) (46).

In the case of enzymes, key requirements include the protein pattern for at least three batches (determined by, i.e., SDS-PAGE analysis or size exclusion chromatography): amino acid sequence, including the calculated molecular mass of the protein, degree of glycosylation (if relevant), and the protein purity. Moreover, the FE activity expressed in units (U) per unit weight should be provided together with the assay used for its measurement. Furthermore, the enzyme optimum values of pH and temperature must be evaluated, together with the FE thermostability. The latter information is fundamental to establishing the degree of residual FE activity under the intended conditions of use. As an example, for the safety assessment of rennet from the abomasum of calves and cows, the enzyme conditions for optimum activity were determined. The rennet milk clotting activity had an optimum pH and temperature of approximately 6.5 and 45°C, respectively. The activity decreased above 50°C, showing no residual activity above 55°C (50).

Moreover, qualitative and quantitative analytical data on inherent substances hazardous to human health should be provided for NF, FE, and FA. Contaminants such as heavy metals (lead, cadmium, mercury, and arsenic), mycotoxins, or pesticides also demand scrutiny and should be in line with the regulated maximum values. For instance, by-products from plant origin should align with Maximum Residue Limits (MRLs) for their specific crop regulation (Regulation (EC) No 396/2005). Quantitative data for heavy metals should be reported in at least three batches of the FE, and their concentrations determined using atomic absorption spectroscopy or inductively coupled atomic-emission spectroscopy, according to JECFA Guidelines.^24^ The general specifications for enzymes used in food processing^23^ stipulate threshold values of 5, 0.5, 0.5, and 3 mg/kg FE for lead, cadmium, mercury, and arsenic, respectively. If known inherent toxic compounds may be present in the source (as assessed by literature search), their concentration in the NF or FE must be analyzed. For instance, the concentrations of phytic acid, ergot alkaloids, patulin, trypsin inhibitors, and lectins from the barley and rice protein as NF were comparable with the occurrence levels of these compounds in other food products and improvement agents (38). Additionally, for the safety assessment of dried coffee husk (cascara) as an NF, the applicant provided data on polycyclic aromatic hydrocarbons (PAHs) such as benzo[*a*]anthracene, benzo[*a*]pyrene, benzo[*b*]fluoranthene, and chrysene, contaminants resulting from the environment or from burning of material (e.g., wood and fossil fuels) in the regions close to the farms, that were below the respective regulatory maximum levels (27).

The stability of NF and FA under the proposed storage conditions should be evaluated and described to identify hazards that might arise during storage and transport. In the case of NF, the data should cover at least the intended shelf-life, and the data requirements may differ depending on the nature of the NF. For instance, when the NF is prone to microbiological deterioration, its microbiological stability during the shelf life has to be investigated, as in the case of beta-lactoglobulin (36). In the case of re-evaluation of L(+)-tartaric acid (E 334) as an FA, it was noted that the stability of the tartrates depends on the alcohol content, pH, and temperature, as well as interactive effects of the solution matrix with the cations (K and Ca) and the bitartrate/tartrate anions. However, the applicant demonstrated its stability for 3 years of storage and compliance with the purity criteria (appearance and physical/chemical parameters) without change in the assay (%) or specific optical rotation (39). In the case of FE, since the shelf-life is out of the assessment scope, no long-term stability data are needed. However, as mentioned above, data on the FE thermal stability is essential to estimate the residual enzyme activity in the final food.

The specifications are a common requirement for NF and FA, and they define the key parameters characterizing the food products and improvement agents that are intended to be placed on the market, as well as the relevant safety compositional parameters and their limits (only for FA). The purity of a single substance needs to be defined by specifications, and adequate chemical characterization of simple mixtures has to be performed.

### Intended uses and use levels and exposure assessment

The data for an assessment of an NF, FE, or FA on the proposed or intended uses should indicate the form of uses of the NF (e.g., whole food and ingredient), FA (liquid and powder formulations), the food categories in which the FA/NF is proposed to be used and intended uses of the FE, and the proposed maximum amounts of the NF in product(s) as consumed or intended use levels of FA/FE.

The safety data and the exposure assessment of NF shall cover all population groups (Commission Implementing Regulation (EU) 2017/2469, article 5). Most of the NF derived from a by-product were proposed for the general population as ingredients in several food categories, such as spent grains from barley (*Hordeum vulgare*) and rice (*Oryza sativa*), beta-lactoglobulin, coffee cascara, rapeseed powder or XOS, or for specific population groups as in the case of betaine as NF (sports people above 10 years of age) or rapeseed powder (individuals above 1 year of age)^25^ (23, 27–29, 33, 36). Information on whether the NF is intended to replace another food and the proposed average and maximum daily intakes for different age/gender groups as appropriate need also to be provided. For FA, for which modification of the proposed uses or use levels is requested, the new proposed use levels, the maximum permitted levels, and the normal use levels of the already authorized uses, if available, should be included. For FE, a flowchart illustrating the raw materials to which the FE is applied and the resulting foods or food ingredients should be provided. In addition, the technological need and function of the FE during the relevant food manufacturing processes should be described.

The evaluation of the safety of the NF is based on the estimation of the anticipated daily intakes (average and high percentiles) for each target population group (including, where relevant, vulnerable groups such as children, pregnant, and lactating women), as well as acute intake when acute effects may be of concern. Different tools are available to applicants to estimate chronic dietary exposure (e.g., EFSA Food Additive Intake Model^26^ and DietEx tool^27^). The highest estimated daily intake (i.e., at least the 95th percentile) among the population groups from a representative database (e.g., EFSA Comprehensive European Food Consumption Database or national dietary surveys) is recommended to be used as the starting point for the safety evaluation. For NF used as food supplements, i.e., shrimp peptide concentrate or egg membrane hydrolysate, the intake was directly derived from the maximum daily use levels proposed by the applicant (34, 35).

In the case of FA, the assessment is based on aggregate exposure from all sources, including its natural occurrence in food, non-additive use in food supplements, use as a nutrient, use as flavoring, use as food contact material, and use in pharmaceutical or cosmetic products. The mean anticipated exposure and high exposure (95th percentile) are requested for all age groups (toddlers, children, adolescents, adults, and elderly). For instance, for the re-evaluation of tartaric acid-tartrates (E 334–337, 354), the exposure included estimates from their use as FA or from related FA and from their natural occurrence as soluble acid in grapes and the principal acid in wine (39).

The dietary exposure assessment to the FE-Total Organic Solid (TOS) arising from the FE production process starts with the revision of the intended uses to consider whether exposure estimates need calculation. Most of the FE derived from animal and plant by-products are intended to be used in milk processing for cheese production (e.g., rennet, triacylglycerol lipase, catalase, and phytopepsin), hydrolysis of milk proteins for use in infant formula and follow on formula (i.e., trypsin), and during baking processing (peroxidase and L-ascorbate oxidase) (49–52, 58, 60–63). A complete dietary exposure assessment is derived by comparison of the FE exposure estimates to the reference point identified from the toxicological studies to calculate a margin of exposure (MoE). However, toxicological testing for FE derived from edible parts of plants or animals may be derogated (see section 3.2.5 and Regulation (EU) No 562/2012). For these FE, the exposure to the FE-TOS is compared to an equal fraction of the source material if proof of consumption of the animal or plant sources is retrieved. The reference to the source of information must be valid and include quantities of consumption within and outside the EU. Furthermore, the enzyme yield factor, i.e., the amount of source material (in kg) necessary to obtain (kg or g) of food enzyme to calculate the dietary exposure to the FE-TOS, should be indicated.

### Toxicological information and absorption, distribution, metabolism, and excretion (ADME)

The toxicological assessments of NF and FA rely on a tiered approach designed to evaluate the (i) toxicokinetics (TK) defined as the study of absorption, distribution, metabolism, and excretion (ADME) in relation to dose/time; (ii) genotoxicity; (iii) subchronic and chronic toxicity; (iv) carcinogenicity; and (v) reproductive and developmental toxicity. FE assessment, instead, requires the use of a standard battery of *in vitro* genotoxicity tests and *in vivo* studies for systemic toxicity unless a specific approach applies (e.g., FE derived from agro-food by-products, as explained below).

All toxicological studies should adhere to international guidelines (e.g., OECD) and Good Laboratory Practices (GLP), forming an integral part of the RA (20–22, 69, 70). Despite the generally recommended classical toxicity testing protocols for products categorized as complex mixtures or whole foods (i.e., dried coffee husk as NF), full compliance with the testing protocols may not be necessary if scientific justification is provided (27).

The genotoxicity of NF and FA is assessed according to the EFSA testing strategy (69). A basic battery of *in vitro* tests is recommended as a first step and in case of positive *in vitro* results, an appropriate *in vivo* study is conducted to assess whether the genotoxic potential observed *in vitro* is expressed *in vivo*. If the product is not a fully characterized mixture, the approach is to test for the genotoxicity of the unidentified fractions or, if not feasible, to perform experimental testing of the whole mixture (71).

Nature and compositional characterization, production process, history of use of the food and its source, and available data retrieved from literature are also taken into consideration during the toxicological assessment. For example, as no concerns arose from the compositional characterization of partially hydrolyzed protein from spent barley (*Hordeum vulgare*) and rice (*Oryza sativa*) (28), the Panel considered that no toxicological studies were needed for these NF. As a special case, botanical FA, derived from conventional food sources with a long-term history of food use, may benefit from a “presumption of safety” under certain circumstances when an adequate body of knowledge exists (21).

The toxicological studies should be conducted with a tested material (e.g., NF or FA) representative of the final formulation unless a rationale is provided for using a different formulation or product. In the case of the shrimp peptide concentrate, for example, the applicant provided studies on genotoxicity, acute oral toxicity, and subchronic toxicity conducted with an intermediate product of the manufacturing process, which differed from the NF only in the peptide size. The Panel considered that it was appropriate for the toxicological test, and no toxicological concerns were raised (34).

Human studies are not required by default, but if they are available, they constitute supporting evidence to demonstrate the safety of by-products, as was the case for the shrimp peptide concentrate and the egg membrane hydrolysate (34, 35).

For FA, such as the soybean hemicellulose, several *in vitro* data indicated that hemicelluloses and its major component (i.e., xylan) are fermented by the gut microbiota, which produces short-chain fatty acids as metabolic by-products. These fermentation products did not raise any safety concerns. Despite the absence of human studies, the Panel considered that hemicellulose, including soybean hemicellulose, is not absorbed but extensively fermented by the intestinal microflora (38).

Toxicologically relevant compounds (e.g., secondary metabolites and naturally occurring contaminants) are also considered from both nutritional and toxicological points of view. The sweetener neohesperidine dihydrochalcone (E 959) may contain impurities, which are degradation products, compounds naturally occurring in the raw materials, or products of side reactions, all closely structurally related to neohesperidine dihydrochalcone. A Q(SAR) analysis by the OECD Q(SAR) Toolbox on the impurities contained in E 959 was performed, indicating no relevant differences between the structural alert profile of E 959 and its impurities and, after assessment, E 959 did not raise genotoxicity concerns (41).

According to the Commission implementing Regulation (EU) 562/2012, the toxicological testing of FE derived from edible parts of non-genetically modified plants and animals, including their by-products, can be waived, provided that the consumption of the source material as such is greater than the potential intake of the FE, and that no (chemical and/or microbiological) hazard is introduced during the manufacturing of the product. For instance, no hazards were introduced during the production of animal-derived FE rennet, which has been used in the production of cheese for centuries; therefore, the need for toxicological testing was waived. Both requirements were met in the case of the plant-derived enzymes L-Ascorbate oxidase from the fruit peel of *Cucurbita pepo* and *Cucurbita moschata* (squash, pumpkins) and for the peroxidase from *Glycine max* (soybean) hulls (62, 66).

When the source of the FE is not commonly consumed (e.g., porcine pancreas-derived FE), a toxicological assessment is normally required. Data obtained by clinical studies with drugs containing pancreatic enzymes of porcine origin and with infant formulae containing protein hydrolysates derived from these enzymes are used for the toxicological evaluation of these types of FE (50). As for any other FE, exemption from toxicity testing is granted when no FE-TOS is carried over in the final foods and proof of its absence is provided.

Altogether, the toxicological approach followed on the different cases of by-products emphasizes that along with the tiered approach for the toxicological RA, the nature and compositional characterization, production process, history of use of the food and its source, human studies, and available data retrieved from literature are of pivotal importance for completing the toxicological screening.

### Allergenicity assessment

The allergenicity assessment of NF, FE, and FA relies on the weight of evidence approach (WoE), which accounts for the cumulative body of available evidence, including scientific literature on the allergenic potential of the product and its source, investigation of structural aspects of the protein or peptide by bioinformatic approaches and homology search of the product (i.e., FE) as compared to known allergens. For NF and FA, *in vitro* and *in vivo* assays and clinical data are also taken into consideration (72–74).

However, predicting whether foods or food improvement agents of a proteinaceous nature may cause an adverse response is challenging due to a lack of complete understanding of the mechanisms underlying immune-mediated reactions. In principle, even traces of allergens present in food may pose a hazard for sensitized individuals. In the context of the safety assessment of NF, FE, and FA, no single method on its own is sufficient to reliably predict the potential of a given protein to sensitize or elicit an allergic reaction. Additionally, no threshold values applicable to allergens for RA purposes are currently available (20–22, 74). In the case of FE, the allergenicity assessment of the food enzyme-TOS can be waived only if proof of its absence from the final food is provided (22).

It is generally assumed that NF-containing proteins to which consumers were potentially never exposed (including peptides) have, by default, allergenic potential (20). According to the EFSA NDA Panel, NF derived from allergenic foods, including processing aids, listed in Annex II of Regulation (EU) No 1169/2011,^28^ should be labeled, as they are assumed to retain the allergenicity potential of the source, regardless of the amounts of final protein content in the NF. This rationale was applied for the safety evaluation of shrimp peptide concentrate (34), spent grains from barley (*Hordeum vulgare*) and rice (*Oryza sativa*) (28), bovine milk basic whey protein isolate (37), and beta-lactoglobulin (BLG) (36). For example, the NDA Panel considered the egg membrane hydrolysate to be potentially allergenic due to its egg origin, despite the negative results when testing the hydrolysates in a radio-allergo-sorbent-inhibition assay and an *in-vivo* sensitization assay on guinea pigs (35). The same consideration was made when the NDA Panel recommended that the BLG produced with soy-lecithin as an emulsifier be labeled as soy allergens (36).

Similarly, the allergenicity assessment of the soybean hemicellulose additive E 426 showcased that several soy proteins have allergenic potential and that a significant number of proteins can be present in the FA under safety evaluation. Therefore, the FAF Panel considered that the quantity of residual proteins in E 426 should be reduced as much as possible. In addition, consumers should be informed of the presence of potentially allergenic proteins in the FA (38).

Regarding FE, according to the methodology provided in the latest guidance (EFSA CEP Panel., 2021) and referred to in FAO/WHO, 2001, the amino acid sequence of the FE should be aligned to the sequence of known allergen(s) to determine its homology. Using a sliding window of 80 amino acids of FE, only sequences with identity >35% to known allergens should be reported. The hits found should be searched in the available literature to discuss their allergenic potential, and if listed in Annex II of Regulation (EU) No 1169/2011, the allergens should be labeled in the marketed product, as explained above for NF and FA. Generally, allergens from the source (i.e., cardoon and soybean) can remain in the FE and represent a risk for allergic reactions in individuals allergic to such source. In the case of the peroxidase extracted from soybean hulls (62), it is considered that soybean proteins may be carried over in the FE, which could determine adverse reactions in soybean-allergic individuals. In addition, the FE is formulated with wheat, which can cause gluten intolerance and food and respiratory allergies in gluten-intolerant and wheat-allergic consumers. Therefore, a comprehensive list of known or putative allergens, which specifically includes those for which mandatory labeling is requested by legislation (14 allergens), should be provided, serving the decision-making by risk managers on the possible labeling of the marketed product.

In cases where the source material and/or the processing aids are not listed in Annex II of Regulation (EU) No 1169/2011, the allergenicity assessment should consider all other available information. Nonetheless, particularly for certain NF derived from by-products, it can be challenging to collect a robust body of evidence, as a thorough characterization of the proteins contained in an NF is required. NF derived from rapeseed (33), coffee (25–27), and cacao (24), as well as betaine produced via yeast fermentation (29), are examples of NF for which the likelihood of allergic reactions was based on such body of evidence.

Regarding FE, any data available in the literature on the enzyme sensitization or elicitation reaction should be evaluated together with information on potential allergenicity caused by enzymes of the same family. For instance, the allergenicity potential of the animal-derived FE rennet, the protease complex containing trypsin, and the phospholipase A2 has been assessed in several EFSA outputs, in which the CEP Panel considered that occupational respiratory allergies and sensitization to dust of these abomasum and porcine pancreas enzymes have been described but are not reported as food allergens (44, 49). Although the FE phospholipase A2 derived from the porcine pancreas is considered the major allergen of honeybee venom, the oral intake of this enzyme was shown to be not of concern. Therefore, the risk of allergic reactions for these FE was considered to be low under the intended conditions of use (46).

Altogether, the allergenicity assessment followed by EFSA on the evaluation of NF, FE, and FA derived from animal and plant by-products is based on similar criteria.

## Differences among the scientific requirements for the safety assessment of novel foods, food enzymes, and food additives derived from by-products

As previously mentioned, specific requirements for the safety assessment of NF, FE, or FA may apply, depending on its intended use. Regarding NF, additional requirements refer to its history of safe use or its source and nutritional assessment.

### History of use of the NF and/or its source

The history of consumption of an NF derived from a by-product or its source has to be investigated by means of data collection available in the public domain, considering both their food and non-food uses. Applicants should perform a comprehensive literature review, when possible, in line with the EFSA principles on such reviews (75) and provide information reporting safety-related aspects on the consumption of the NF or similar foods, including full reports of human studies when available. Such data may be relevant for concluding the safety of an NF, especially in the case of whole foods, for which toxicological studies might be challenging to perform due to the nature of the NF itself. For instance, in the safety assessment of dried coffee husk (cascara) as an NF, a by-product from *Coffea arabica* L., due to (i) the long history of safe use of the fruit and of the cascara *per se* in countries outside the EU and (ii) a thorough product characterization of the NF, EFSA could conclude positively on the safety of the NF, under the proposed conditions of use, without further toxicological studies (27).

### Nutritional assessment of the NF

The nutritional content of the NF should be evaluated, alongside the proposed uses and use levels, to investigate whether the NF can be nutritionally disadvantageous for consumers, i.e., has the potential to cause nutritional inadequacy or excess, at the anticipated levels of intake (20). The information required refers to the nutrients and antinutrients present in the NF and addresses their bioavailability, considering the effects of the manufacturing processes and storage. For instance, when assessing the safety of egg membrane hydrolysate as an NF, the alkaline treatment conditions employed during the manufacturing process resulted in partial racemization of the amino acids in the NF, thus limiting the digestibility/bioavailability of such amino acids. However, considering the proposed conditions of use (i.e., 450 mg/day), it was concluded that the NF was not nutritionally disadvantageous (35). In addition, due to the sources of those NF, information on antinutrients (i.e., tannins, trypsin inhibitors, amylase inhibitors, phytic acid and phytates, and oxalates or saponins) in the spent grains from barley (*Hordeum vulgare*) and rice (*Oryza sativa*), coffee husk, and rapeseed powder from *Brassica rapa* outputs were provided (27, 28, 33).

The levels of use and estimated intakes for the target population should be considered, as explained above in section 4.2.4., by discussing the intakes taking into account the dietary reference values (DRVs), including tolerable upper intake levels (ULs). The estimations from the NF consumption and the usual intake from the background diet are compared with the ULs of nutrients (if available) to assess the risk of excess intake (20, 76, 77). For assessing the risk of excessive iron intake in the assessment of whey basic protein isolate that contains lactoferrin, an iron-binding glycoprotein, EFSA concluded that the estimated values for the population groups were below the DRVs for iron (37). In the safety assessment of traditional food from third countries (i.e., pulp from *Theobroma cacao* L), a nutritional assessment is not necessarily needed (78).

## Discussion, ongoing trends, and future perspectives

The utilization of agro-food by-products for the production of new food products, such as NF, and food improvement agents, including FA and FE, reflects an integrated effort toward a more sustainable food system. The safety of such products must be assessed by EFSA, when applicable, before any FBO can market them on the EU market. EFSA’s commitment to scientific excellence and transparency ensures that food products and food improvement agents derived from by-products undergo rigorous scrutiny, also fostering a regulatory environment that encourages sustainable practices and innovation within the food industry. As the review outlines, the utilization of by-products falls under different regulatory frameworks, depending on the intended uses, such as NF, FE, or FA. FBOs are advised to adhere to the requirements outlined in the EFSA Guidance documents to prepare their dossiers. The scientific prerequisites to allow for the evaluation of their safety are similar among the different Guidance documents, with similar information needed in terms of identity, production process, compositional characterization, proposed/intended uses and exposure assessment, toxicological information, and allergenicity data. For NF, additional data are required to inform the potentially existing history of safe use of the NF or its source, and nutritional assessment is needed to investigate whether an NF is nutritionally disadvantageous for consumers. Additionally, information on existing authorization and evaluations is needed in the case of FA and NF. A growing trend is observed in using by-products to produce novel foods and food improvement agents. Currently, EFSA has ongoing safety assessments for NF derived from carrot pomace, water olive mill by-products, and chitosan powder; FA derived from brans and husks of various plant seeds, fibers extracted from seed pods, or peels of fruits or vegetables; and FE derived from the abomasum of cows, goats, and sheep, as well as other sources such as the pancreas of pigs, papaya fruit peels, and pineapple stems. This study showcases the approaches for the safety evaluation of NF, FE, and FA derived from by-products and outlines the similarities and differences among the different EFSA Guidance documents, which could raise the awareness of the FBOs for the scientific data requirements of the applications, helping them in preparing well-structured dossiers. It is important to mention that harmonization of regulations is outside EFSA’s remit; therefore, the topic was not addressed in this study. It is also worth noting that to keep its risk assessment fit for purpose, EFSA is constantly updating its guidance documents.

Moreover, the EFSA’s Strategy 2027 tackles future challenges by emphasizing coordinated assistance to the EC sustainability agenda. This includes preparedness to further develop methodologies to identify emerging risks at the global level and suggests prevention strategies to safeguard the safety and sustainability of food systems (2). As an example, EFSA organized a scientific colloquium in May 2023 on “Cell Culture-derived Foods and Food Ingredients” that aimed to identify relevant stakeholders, review state-of-the-art on the respective topics, and discuss emerging safety and methodological aspects and their impact on EFSA’s risk assessment approaches (79). Furthermore, EFSA, together with its sister agencies,^29^ is committed to a One Health approach that aims to optimize the health of people, animals, and ecosystems. This translates through addressing challenges such as zoonotic and (re-)emerging infectious diseases, non-communicable diseases linked to environmental risk factors, antimicrobial resistance (AMR), and climate change mitigation (80–82). For instance, in the context of antimicrobial resistance, the incorporation of the gut microbiome in food safety risk assessment of xenobiotics is also investigated to elucidate their involvement in the balance between health and disease (83, 84). Additionally, the use of alternative tools, such as New Approach Methodologies, is also investigated for the new risk assessment approaches, fostering a future with less or no animal testing in food safety (85–87).

Finally, EFSA is committed to further strengthening an open and transparent dialog with the food chain stakeholders through a dedicated framework^30^ that includes a registration option for organizations that are willing to engage more closely with EFSA. Collaboration with stakeholders is key to identifying emerging issues requiring EFSA’s preparedness and supporting EFSA’s scientific risk assessment with relevant evidence and information.

## Author contributions

GP: Writing – review & editing, Writing – original draft, Visualization, Validation, Project administration, Methodology, Investigation, Data curation, Conceptualization. EM: Writing – review & editing, Writing – original draft, Visualization, Validation, Project administration, Methodology, Investigation, Data curation, Conceptualization. PZ: Writing – review & editing, Writing – original draft, Visualization, Validation, Project administration, Methodology, Investigation, Data curation, Conceptualization. EB: Writing – original draft, Data curation. CC: Writing – original draft, Data curation. CF-F: Writing – review & editing. EG: Writing – original draft. ML: Writing – original draft, Data curation. MM: Writing – original draft, Data curation. AM: Writing – original draft, Data curation. EN: Writing – original draft, Data curation. IN: Writing – original draft, Data curation. PR: Writing – original draft, Visualization, Data curation. RR: Writing – original draft, Data curation. AR: Writing – review & editing. LR: Writing – review & editing, Data curation. FS: Writing – review & editing, Writing – original draft, Data curation. EV: Writing – review & editing, Data curation. YL: Writing – review & editing, Supervision. CS: Writing – review & editing, Supervision. AG: Writing – review & editing, Supervision.

## Footnotes

^1^Regulation (EU) 2015/2283 of the European Parliament and of the Council of 25 November 2015 on novel foods, amending Regulation (EU) No 1169/2011 of the European Parliament and of the Council and repealing Regulation (EC) No 258/97 of the European Parliament and of the Council and Commission Regulation (EC) No 1852/2001.

^2^Regulation (EC) No 1333/2008 of the European Parliament and of the Council of 16 December 2008 on food additives.

^3^Regulation (EC) No 1332/2008 of the European Parliament and of the Council of 16 December 2008 on Food Enzymes and Amending Council Directive 83/417/EEC, Council Regulation (EC) No 1493/1999, Directive 2000/13/EC, Council Directive 2001/112/EC, and Regulation (EC) No 258/97. OJ L 354, 31.12.2008, pp. 7–15.

^4^Regulation (EC) No 258/97 of the European Parliament and of the Council of 27 January 1997 concerning novel foods and novel food ingredients.

^5^Commission Implementing Regulation (EU) 2018/456 of 19 March 2018 on the procedural steps of the consultation process for determination of novel food status in accordance with Regulation (EU) 2015/2283 of the European Parliament and of the Council on novel foods.

^6^Commission Implementing Regulation (EU) 2017/2470 of 20 December 2017 establishing the Union list of novel foods in accordance with Regulation (EU) 2015/2283 of the European Parliament and of the Council on novel foods.

^7^Regulation (EC) No 1331/2008 of the European Parliament and of the Council of 16 December 2008 establishing a common authorization procedure for food additives, food enzymes, and food flavorings. OJ L 354, 31.12.2008, pp. 1–6.

^8^Commission Regulation (EU) No 234/2011 of 10 March 2011 implementing Regulation (EC) No 1331/2008 of the European Parliament and of the Council establishing a common authorisation procedure for food additives, food enzymes, and food flavorings. OJ L 64, 11.3.2011, pp. 15–24.

^9^Commission Regulation (EU) No. 1130/2011 amending Annex III to Regulation (EC) No. 1333/2008 of the European Parliament and of the Council on food additives by establishing a Union list of food additives approved for use in food additives, food enzymes, food flavorings, and nutrients.

^10^Commission Regulation (EU) No 231/2012 of 9 March 2012 laying down specifications for food additives listed in Annexes II and III to Regulation (EC) No 1333/2008 of the European Parliament and of the Council.

^11^Food Additives (European Food Safety Authority). Retrieved from https://www.efsa.europa.eu/en/topics/topic/food-additives.

^12^Commission Regulation (EU) No 257/2010 of 25 March 2010 setting up a program for the re-evaluation of approved food additives in accordance with Regulation (EC) No 1333/2008 of the European Parliament and of the Council on food additives.

^13^Commission Implementing Regulation (EU) No 562/2012 of 27 June 2012 amending Commission Regulation (EU) No 234/2011 with regard to specific data required for risk assessment of food enzymes.

^14^ https://efsa.onlinelibrary.wiley.com/journal/18314732

^15^Regulation (EC) No 396/2005 of the European Parliament and of the Council on maximum residue levels of pesticides in or on food and feed of plant and animal origin and amending Council Directive 91/4141/EEC.

^16^Commission Regulation (EU) No 2015/1162 of 15 July 2015 amending Annex V to Regulation (EC) No 999/2001 of the European Parliament and of the Council laying down rules for the prevention, control and eradication of certain transmissible spongiform encephalopathies.

^17^Regulation (EC) No 1774/2002 of the European Parliament and of the Council of 3 October 2002 laying down health rules concerning animal by-products not intended for human consumption.

^18^Regulation (EC) No 852/2004 of the European Parliament and of the Council of 29 April 2004 on the hygiene of foodstuffs. OJL 139, 30.4.2004, pp. 54.

^19^Regulation (EC) No 853/2004 of the European Parliament and of the Council of 29 April 2004 laying down specific hygiene rules for food of animal origin. OJ L226, 25.6.2004, p. 22.

^20^Directive 2009/32/EC of the European Parliament and of the Council of 23 April 2009 on the approximation of the laws of the Member States on extraction solvents used in the production of foodstuffs and food ingredients. OJ L 141, 6.6.2009, p. 3–11.

^21^Commission Notice on the implementation of food safety management systems covering prerequisite programs (PRPs) and procedures based on the HACCP principles, including the facilitation/flexibility of the implementation in certain food businesses. C/2016/4608.

^22^Regulation (EC) No 852/2004 of the European Parliament and of the Council of 29 April 2004 on the hygiene of foodstuffs.

^23^By-product obtained from fermentation where molasses are used as a carbon source.

^24^FAO/WHO (Food and Agriculture Organization of the United Nations/World Health Organization), 2006. General specifications and considerations for enzyme preparations used in food processing in Compendium of food additive specifications. 67th meeting. FAO JECFA Monographs, 3, 63–67. Available online: http://www.fao.org/ 3/a-a0675e.pdf

^25^Even if the NF is intended to be used as an ingredient in standard food categories, it can be consumed by any group of the population. Therefore, the safety data and the exposure assessment shall cover all population groups Commission implementing Regulation (EU) 2017/2469, article 5.

^26^ https://www.efsa.europa.eu/en/applications/food-improvement-agents/tools

^27^ https://www.efsa.europa.eu/ro/science/tools-and-resources/dietex

^28^Regulation (EU) No 1169/2011 of the European Parliament and of the Council of 25 October 2011 on the provision of food information to consumers.

^29^European Chemicals Agency (ECHA); European Environment Agency (EEA) and European Medicines Agency (EMA).

^30^ https://www.efsa.europa.eu/en/engage/stakeholders
