# Supplementary material for: Novel foods, food enzymes, and food additives derived from food by-products of plant or animal origin: principles and overview of the EFSA safety assessment
Source: Front Nutr. 2024 May 3;11:1390734. doi: 10.3389/fnut.2024.1390734 (PMC11165998; doi:10.3389/fnut.2024.1390734)
Supplement: Supplementary file 1 [file Table_1.docx]

Supplementary Material

Novel foods, food enzymes and food additives derived from food by-products of plant or animal origin. Principles and overview of the EFSA safety assessment

**Gabriela Precup^1(†)^, Eleonora Marini^3(†)^, Panagiota Zakidou^2(†),^ Elisa Beneventi^1^, Civitella Consuelo^2^, Cristina Fernández-Fraguas^3,^ Esther Garcia Ruiz^1^, Marcello Laganaro^1^, Maura Magani^1^, Agnieszka Mech^2^, Estefania Noriega Fernandez^1,4,5^, Irene Nuin Garciarena^1^, Pablo Rodriguez Fernandez^1^, Ruth Roldan Torres^1^, Annamaria Rossi^1^, Laura Ruggeri^2^, Francesco Suriano^1,6^, Ermolaos Ververis^1,7^, Yi Liu^3(†),^ , Camilla Smeraldi^2(†)^, Andrea Germini^1(†)^**

^1^ European Food Safety Authority, Nutrition and Food Innovation Unit, Novel Foods Team, Parma, Italy

^2^ European Food Safety Authority, Food Ingredients and Packaging Unit, Food Additives and Flavourings Team, Parma, Italy

^3^ European Food Safety Authority, Food Ingredients and Packaging Unit, Food Enzymes Team, Parma, Italy

^4^ Nofima, Department of Processing Technology. Richard Johnsens gate 4, 4021 Stavanger, Norway

^5^ University of Zaragoza, Faculty of Veterinary, Department of Animal Production and Food Science, Food Technology Group. Miguel Servet 177. 50013, Zaragoza, Spain

^6^ Department of Medical Biochemistry and Cell Biology, Institute of Biomedicine, University of Gothenburg, Gothenburg, Sweden

^7^ Department of Hygiene, Epidemiology and Medical Statistics, School of Medicine, National and Kapodistrian University of Athens, Greece

*** Correspondence:** Corresponding Author: [gabriela.precup@efsa.europa.eu](mailto:gabriela.precup@efsa.europa.eu); [NIF@efsa.europa.eu](mailto:NIF@efsa.europa.eu)

†These authors have contributed equally to this work and share first authorship

†These authors have contributed equally to this work and share last authorship

**Supplementary Table 1. Overview of the potential hazards rising from the source and production process of novel foods, food enzymes and food additives and their assessment**

|  | **By-product** | **Product of interest** | **Regulatory category** | **Production process principle** | **Potential hazards from the source and the production process** | **Critical findings** | **Reference** |
| --- | --- | --- | --- | --- | --- | --- | --- |
| 1. | Corncobs (*Zea mays* subs. *mays*) (non-GMO) | XOS | NF | Enzymatic reaction (hydrolysis) | Contaminants from the raw material (heavy metals, pesticides, mycotoxins) | Negative PCR on corncobs, aflatoxins, heavy metals and pesticides <LOQs | 23 |
|  |  |  |  |  | Use of enzyme from a non QPS microorganism (capacity to produce peptaibols, antimicrobial peptides, other compounds with unknown biological activity (EFSA BIOHAZ Panel, 2013). | Enzyme compiled with JEFCA requirements for food enzymes (JEFCA, 2006)  No antibacterial activity  No presence of peptaibols in the FE in amounts that would raise safety concern. |  |
|  |  |  |  |  | Residual enzymatic activity | Xylanase activity below the limit of detection (10 U/g) of the applied assay |  |
| 2. | Spent grains from barley (*Hordeum vulgare*) and rice *Oryza sativa*) | Partially hydrolysed protein | NF | Enzymatic reaction (hydrolysis) | Use of enzymes from GM strains | Absence of recombinant DNA absence of viable cells of the production microorganisms demonstrated according to EFSA CEP Panel, 2021 | 28 |
| 3. | De-oiled press-cake from rapeseeds (*Brassica rapa* L. and *Brassica napus* L, non-GM double low (00) cultivars) | Rapeseed powder | NF | Chemical and enzymatic reaction (water-ethanol extraction and enzymatic hydrolysis) | Contaminants from the raw material (heavy metals, pesticides, mycotoxins) | Pesticide residues and heavy metals monitored; Botanical impurities (< 3%), chlorophyll content (< 50 ppm), free fatty acids (< 2% as oleic acid), moisture (< 9%) | 33 |
|  |  |  |  |  | Residual enzymatic activity | DM Food grade 3-phytase-MaxamylTM from *Aspergillus niger* – Registered under the REACH Regulation (ECHA, 2019), authorised as a feed additive (EFSA. 2006; EFSA FEEDAP Panel, 2019), evaluated by JECFA with no safety concerns (Choudhuri et al., 2012) |  |
| 4. | Molasses, vinasses, betaine-glycerol from sugar beet (*Beta vulgaris* L.), Non-GM | Betaine | NF | Chemical reaction (water extraction) | Potential presence of *Saccharomyces cerevisiae* (baker’s yeast) in the final NF from the non-GM sugar beet vinasses | *S. cerevisiae* removed from final NF by control steps (e.g. microfiltration) | 29 |
| 5. | Coffee husk (cascara) from Coffee (*Coffea arabica* L) | Coffee husk (cascara) | NF | Physical separation (Drying) | Pesticides residue levels from the raw material | Most pesticide levels <LOD of the analytical method employed, except zoxystrobin, cyproconazole, epoxiconazole, tebuconazole and triadimenol- concentrations higher than the MRL set for coffee bean in EU but lower than the MRL set for other foodstuffs by EU Regulations. | 27 |
| 6. | Coffee cherry pulp (*Coffea arabica* L) | Coffee cherry pulp | TF | Physical separation (Drying) | Contaminants from the raw material (heavy metals, pesticides, mycotoxins) | Heavy metals < LOD; pesticides Pesticide levels comply with Regulation (EC) No 396/2005 for ‘0639000’ for ‘Herbal infusions from any other parts of the plant’  Mycotoxins < LOD of the analytical method employed. | 25 |
| 7. | Cocoa pulp from Cocoa fruits (*Theobroma cacao* L) | Cocoa pulp | TF | Chemical reaction (pasteurization) | Presence of biological hazards in the raw material | Biological hazards are controlled via freezing and pasteurisation | 24 |
| 8. | Soybean (*Glycine max*) hulls | Peroxidase | FE | Extraction (alkaline hydrolysis) | Potential contaminants from the raw material (heavy metals, pesticides, mycotoxins) | Quality of the raw materials is regularly controlled: heavy metals and microbiological criteria within legal requirements.  Mycotoxins < LOD of the respective method. | 62 |
|  |  |  |  |  | Allergenic proteins and other hazards introduced in the manufacturing of the FE | The FE might contain soybean allergens (SDS-PAGE provided); wheat used as a carrier; thus the FE preparation contains wheat allergens and gluten, of safety concerns in wheat-allergic and gluten intolerant consumers |  |
| 9. | Soybean (*Glycine max*) fibre (Okara) | Soybean hemicellulose (E 426) | FA | Extraction (water) | Presence of heavy metals (arsenic, lead, mercury and cadmium), proteins, ethanol, pathogenic microorganisms (i.e., *Escherichia coli)* | Re-evaluated. Proposal to revise the maximum specification limits for the impurities of toxic elements (arsenic, lead, mercury and cadmium) and to reduce the amount of residual proteins in E 426, relevant to its allergenic proteins | 38 |
| 10. | Squash, pumpkins (non-GMO) peels (*Cucurbita pepo* and *Cucurbita moschata)* | L-Ascorbate oxidase | FE | Extraction (water) | Potential contaminants from the raw material (heavy metals, pesticides, mycotoxins) | Favourable opinion. Contaminants and pesticides residues within legal requirements. | 67 |
|  |  |  |  |  | Potential hazards introduced during the manufacturing process of the FE | Manufacturing process considered as not of concern |  |
| 11. | Cardoon Pistils (*Cynara cardunculus*) ^a^ | Phytepsin (plant coagulant) | FE | Extraction (water) | Potential contaminants from the raw material (heavy metals, pesticides, mycotoxins) | Favourable opinion. Contaminants and pesticides residues within legal requirements | 63 |
|  |  |  |  |  | Potential hazards introduced in the manufacturing of the FE | Manufacturing process considered as not of concern |  |
| 12. | Tartar, lees and grape marc of wine and/or grape pomace | L(+)-Tartaric acid | FA | Chemical reaction (alkaline and acidic hydrolysis) | Toxic elements resulting from the use of any catalyst, (e.g. vanadium, molybdenum and tungsten) | Re-evaluated. Specifications to be amended to include more information on the manufacturing processes, and limits for toxic elements (e.g. vanadium, molybdenum and tungsten) resulting from the use of catalysts | 39 |
|  |  |  |  |  | Presence of *Rhodococcus ruberstrain* CM001 or *Rhodococcus* sp. strain USA-AN012) | Absence of the strains and their DNA |  |
| 13. | Peat, wood, cellulose residues, coconut shells or other shells) | Vegetable carbon (E 153) | FA | Steam activation | Polycyclic aromatic hydrocarbons (PAHs) and toxic elements arsenic, lead, mercury, cadmium, aluminium, nanoparticles | Re-evaluated. Specifications to be amended to include a requirement for residual carcinogenic PAHs expressed as benzo[a]pyrene using a validated analytical method of appropriate sensitivity (e.g. with a LOD of 0.1 μg/kg), a maximum level of aluminium, and a restriction of the particle size (below 100 nm) in order to exclude the presence of nanoparticles. | 40 |
| 14. | Bitter orange (*Citrus aurantium*) and Grapefruit (*Citrus paradisii*) peels | Neohesperidine dihydrochalcone (E 959) | FA | Chemical extraction (hydroalcoholic) | Structurally related flavonoid impurities, arsenic, lead | Re-evaluated. Derived ADI of 20 mg/kg bw per day for E 959, proposal to lower the current specification limits for arsenic and lead | 41 |
| 15. | Bovine whey (*Bos taurus*) | Galactooligosaccharides (GOS) b | NF | Enzymatic reaction (hydrolysis) | Not mentioned | “The Panel notes that an assessment of the production process for GOS when produced by microbial b-galactosidases was conducted by FSAI in 2013 (FSAI, 2013). This opinion supported the substantial equivalence with Vivinal®, GOS already permitted for use in foods. The production process has not changed since then.” | 30-32 |
|  |  | Beta-lactoglobulin (BLG) | NF | Chemical reaction (crystallization) | Potential presence of contaminants (heavy metals, aflatoxins) | Heavy metals and mycotoxins < LOD of the analytical method employed | 36 |
| 16. | Porcine pancreas | Protease complex containing trypsin^c^ | FE | Extraction | Potential contaminants in the raw material. | Favorable opinion.  FE tested for heavy metals, total coliforms, hepatitis E virus and antimicrobial activity. All parameters within legal requirements. | 42 |
|  |  |  |  |  | Potential hazards introduced in the manufacturing of the FE | Manufacturing process considered as not of concern |  |
|  |  | Phospholipase A2 | FE | Extraction (alkaline and acidic) | Potential contaminants in the raw material. | Inconclusive opinion. Insufficient information about the food enzyme manufacturing process, chemical characterisation of the food enzyme, and incomplete purity data (no equivalence to Food Hygiene Regulation (EC) No 846) | 46 |
|  |  |  |  |  | Potential hazards introduced in the manufacturing of the FE |  |  |
| 17. | Porcine liver | Catalase | FE | Confidential method | Potential contaminants in the raw material. | Unfavorable opinion. FE not in compliance with EU safety requirements. | 60 |
|  |  |  |  |  | Potential hazards introduced in the manufacturing of the FE | Not permitted organic solvent used in the extraction of the FE |  |
| 18. | Abomasum of cows, goats and sheeps (adults and suckling) ^e^ | Animal Rennet | FE | Extraction | Potential contaminants in the raw material. | Favorable opinion. The abomasum of calves and cows is considered fit for human consumption and is an edible offal as defined in Regulation (EC) No 853/2004  FE tested for lead, total coliforms, *Escherichia coli* STEC, *Campylobacter* spp. and yeast and fungi. All parameters within legal requirements. | 50 |
|  |  |  |  |  | Potential hazards introduced in the manufacturing of the FE | Manufacturing process considered as not of concern |  |
| 19. | Pregastric tissues (gullet) of cattle, goat and sheep | Triacylglycerol lipase | FE | Extraction | Potential contaminants in the raw material. | Favorable opinion. The pregastric tissues of calves, young goats and lambs are considered fit for human consumption. It is an edible offal as defined in Regulation (EC) No 853/2004. | 58 |
|  |  |  |  |  | Potential hazards introduced in the manufacturing of the FE | Manufacturing process considered as not of concern  The FE complies with EFSA requirenments for chemical composition, activity and purity. |  |
| 20. | Cattle or pigs blood | Thrombin | FE | Protein isolation and purification by ion-exchange chromatography | Potential contaminants in the raw material. | Favorable opinion. Blood is hygienically collected in approved EU slaughterhouses and treated in accordance with good hygienic practices. Absence of any risk of infectivity (including transmissible spongiform encephalopathies provided). | 59 |
|  |  |  |  |  | Potential hazards introduced in the manufacturing of the FE. | Thrombin is produced under hygienic conditions in accordance with Regulation (EC) No 853/2004 to process meat. |  |
| 21. | Egg membrane | Egg membrane hydrolysate | NF | Chemical reaction (hydrolysis) | Formation of lysinoalanine and partial racemisation of amino acids in the NF due to the conditions employed during the manufacturing process (heat treatment under alkaline pH) | Taking into account the proposed daily intake of the NF (i.e. 450 mg) and considering the dietary background intake from other processed protein-based foods, the potential intake of lysinoalanine or D-amino acids via the NF is not considered to be of safety concern | 35 |
| 22. | Shrimp (*Pandalus borealis*) peptide concentrate | Shrimp shells and heads | NF | Chemical reaction (Hydrolysis) | Residual enzymatic activity | The enzyme (protease) is obtained from non-pathogenic and non-toxicogenic *Bacillus licheniformi*s and/or *Bacillus amyloliquefaciens* and complies with the recommended purity specifications for food enzymes given by the Joint FAO/WHO Expert Committee on Food Additives (JECFA, 2006) and the Food Chemicals Codex (FCC, 201) However, since the NF is purified (membrane filtration , ultrafiltration with a molecular weight (MW) cut-off of 1 kDa), it is envisaged that only small peptides to be present. It is also noted that 99.9% of peptides in NF have a MW<2 kDa. | 34 |
|  |  |  |  |  | Growth of pathogenic bacteria | The temperature of the hydrolysate is kept >72°C to eliminate the growth of pathogenic bacteria |  |

a: Example of one of the four outputs on phytepsin from Cardoon Pistils

b: Example of one of the three outputs on galactooligosaccharides (GOS) from bovine whey

c: Example of one of the six outputs on trypsin from porcine pancreas

d: Example of one of the nine outputs on animal rennet from abomasum of cows, goats and sheeps (adults and suckling)
